# Comparative Evaluation of MaxQuant and Proteome Discoverer MS1-Based Protein Quantification Tools

**DOI:** 10.1021/acs.jproteome.1c00143

**Published:** 2021-05-26

**Authors:** Antonio Palomba, Marcello Abbondio, Giovanni Fiorito, Sergio Uzzau, Daniela Pagnozzi, Alessandro Tanca

**Affiliations:** † Porto Conte Ricerche, Loc. Tramariglio, 07041 Alghero, Italy; ‡ Department of Biomedical Sciences, University of Sassari, Viale San Pietro 43/B, 07100 Sassari, Italy; § MRC Centre for Environment and Health, Imperial College London, Norfolk Place, W2 1PG London, U.K.

**Keywords:** accuracy, differential analysis, label-free quantification, log ratio, mass spectrometry, precision, proteomics, sensitivity, specificity

## Abstract

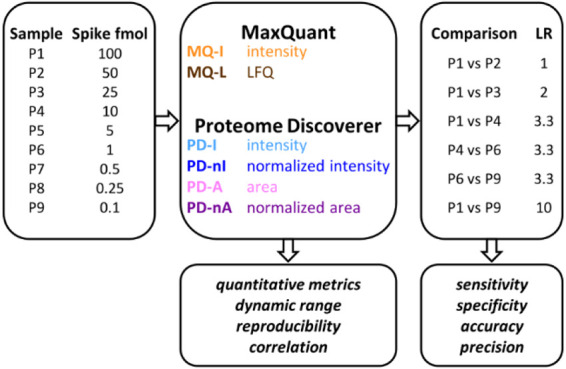

MS1-based label-free quantification can compare precursor ion peaks across runs, allowing reproducible protein measurements. Among bioinformatic platforms enabling MS1-based quantification, MaxQuant (MQ) is one of the most used, while Proteome Discoverer (PD) has recently introduced the Minora tool. Here, we present a comparative evaluation of six MS1-based quantification methods available in MQ and PD. Intensity (MQ and PD) and area (PD only) of the precursor ion peaks were measured and then subjected or not to normalization. The six methods were applied to data sets simulating various differential proteomics scenarios and covering a wide range of protein abundance ratios and amounts. PD outperformed MQ in terms of quantification yield, dynamic range, and reproducibility, although neither platform reached a fully satisfactory quality of measurements at low-abundance ranges. PD methods including normalization were the most accurate in estimating the abundance ratio between groups and the most sensitive when comparing groups with a narrow abundance ratio; on the contrary, MQ methods generally reached slightly higher specificity, accuracy, and precision values. Moreover, we found that applying an optimized log ratio-based threshold can maximize specificity, accuracy, and precision. Taken together, these results can help researchers choose the most appropriate MS1-based protein quantification strategy for their studies.

## Introduction

MS1-based methods are widely used for protein quantification in shotgun proteomics, in view of their flexibility and cost-effectiveness.^1,2^ Using these approaches, a peptide can be successfully quantified across all samples of a data set, even if identified by MS2 in a single sample. This can be pursued by detecting and comparing MS1 ion current peaks of that peptide across runs, thus allowing reproducible protein measurements and minimizing missing values (MVs).^3^ One of the most used tools for MS1-based quantitative proteomics, MaxQuant (MQ), employs a “match between runs” function, able to match precursor ion *m*/*z* and peak retention time information among different sample runs and to infer the peptide identity from run(s) with a valid MS2 identification.^4^ The Proteome Discoverer (PD) platform from Thermo Fisher Scientific has recently introduced the “Minora Feature Detector” node, designed as well to perform an efficient MS1-based quantification by detecting, aligning, and matching peaks across LC/MS runs, and by mapping them to the corresponding peptide sequences identified by MS2. This tool can provide two types of quantitative MS1-based measures, namely the height of the most abundant peak at the apex of the chromatographic profile (“intensity”) or the integrated peak area (“area”). To date, no studies evaluated the performance of PD’s Minora in comparison with other state-of-the-art protein quantification tools.

Nonbiological variability of MS data is due to many factors, including sample preparation and instrumental biases. Both MQ and PD can carry out a chromatogram alignment step, aimed to minimize variability in LC retention time. Several postprocessing methods for label-free quantitative data normalization have also been proposed to reduce systematic biases and, therefore, increase robustness of downstream statistical analyses.^5,6^ To this regard, MQ is able to apply an optimized normalization strategy to the intensity profile, providing an “LFQ” quantitative value (in addition to the “raw” intensity value).^7^ PD users have as well the opportunity to apply a data normalization step, which can be based on total peptide intensity or on the abundance of an internal reference protein.

Here, we comparatively evaluated the performance of six MS1-based label-free protein quantification methods available in the MQ and PD suites, based on different measures (namely, intensity or area of the precursor ion peak) and including or not a normalization step. To this aim, we reanalyzed a previously published proteomic data set where a mix of human standard proteins was spiked at varying amounts in a yeast lysate background. This allowed us to simulate various differential proteomics experimental settings, covering a wide range of protein abundance ratios and amounts. Differential analysis results obtained with the six quantification methods were compared in terms of sensitivity, specificity, accuracy, and precision. A second data set containing a “blank” sample (background only) was also analyzed to further investigate the performance of the compared methods.

## Experimental Section

### Data Sets and MS Analysis Parameters

The main part of the study is a reanalysis of a proteomic data set deposited on the ProteomeXchange repository with the identifier PXD001819 and described by Ramus and colleagues.^8^ The data set had been obtained by spiking a proteomic standard composed of an equimolar mixture of 48 human proteins (UPS1) at nine different amounts into a background (2 μg) of yeast cell lysate. According to the original paper, LC–MS/MS analyses were performed using a nanoRS UHPLC system (Dionex, Amsterdam, The Netherlands) coupled to an LTQ-Orbitrap Velos mass spectrometer (Thermo Fisher Scientific, Bremen, Germany). Samples were loaded on a C-18 precolumn (300 μm ID × 5 mm) at 20 μL/min using a 5% acetonitrile (ACN), 0.05% trifluoroacetic acid solution. After desalting, peptides were separated in an analytical C-18 column (75 μm ID × 15 cm), equilibrated in 95% solvent A (5% ACN, 0.2% formic acid (FA)) and 5% solvent B (80% ACN, 0.2% FA). Peptide elution was carried out at 300 nL/min flow rate, using a solvent B gradient as follows: 5 to 25% for 75 min; 25 to 50% for 30 min; 50 to 100% for 10 min. The mass spectrometer was operated in data-dependent acquisition mode using the XCalibur software. Survey scans were acquired in the Orbitrap on the 300–2000 *m*/*z* range, with resolution set to 60 000. The 20 most intense ions per survey scan were selected for CID fragmentation and the resulting fragments were analyzed in the LTQ. Dynamic exclusion was set to 60 s. A total of 27 raw files, corresponding to nine different amounts of UPS1 proteins (namely 100, 50, 25, 10, 5, 1, 0.5, 0.25, and 0.1 fmol, named P1–P9) analyzed in triplicate, were retrieved from the ProteomeXchange repository and reanalyzed in this study.

A second data set, deposited in the ProteomeXchange repository with identifier PXD003472 and described by Jarnuczak and colleagues,^9^ was selected to further investigate method specificity. Within the data set, we specifically choose two samples, both containing 500 ng of yeast protein digest as background: the first one (YH) with 25 fmol of spiked-in human proteins (the same UPS1 standard used in the main data set); the second one (Y) with no spiked-in proteins (background proteins only). Four replicate runs were available for each sample. According to the original paper, LC-MS/MS analyses were carried out using a nanoAcquity UPLC system (Waters, Manchester, UK) coupled with an LTQ-Orbitrap Velos mass spectrometer. Peptide mixtures were loaded on a 75 μm × 25 cm, 1.8 μm particle size, C18 nanoAcquity analytical column in mobile phase A (0.1% FA) and separated with a linear gradient of 3–35% mobile phase B (0.1% FA in ACN) at a flow rate of 300 nL/min over a 240 min gradient. The instrument was operated in a data-dependent mode. A survey scan was acquired over the 350–2000 *m*/*z* range at a mass resolution of 30 000 and the top 20 most intense precursor ions were subjected to CID fragmentation.

A complete list of MS parameters, retrieved from the deposited raw files of the two data sets, is provided in Data Set S1.

### Protein Identification and Quantification

Protein identification and quantification were performed on the whole data set using two search engine platforms: MaxQuant (MQ; version 1.6.0.13)^10^ and Proteome Discoverer (PD; version 2.4.1.15; Thermo Fisher Scientific). A given protein was considered as “identified” when a valid MS2 spectrum was available for at least one of the peptides belonging to that protein. Quantification was carried out according to six different methods. MQ protein quantification returned a (raw) intensity value (MQ-I) and an “LFQ” (normalized intensity) value (MQ-L). PD provided for each quantified protein the height of the most abundant peak at the apex of the chromatographic profile (“intensity”) and the integrated peak area (“area”); furthermore, quantitative values were subjected (or not) to a normalization step, based on the total peptide intensity of the samples. Therefore, the four PD-based quantitative methods were named intensity (PD-I), normalized intensity (PD-nI), area (PD-A), and normalized area (PD-nA). The quantification value reported for a given protein is calculated as the sum of the quantification values of all peptides belonging to that protein.

A complete list of the parameters used in the MQ and PD protein identification and quantification processes is provided in Data Set S2. Specifically, the time windows for chromatographic peak alignment and matching/mapping were set for both platforms at 10 and 2 min, respectively, based on the default or automatically calculated values reported by PD. Moreover, False-Discovery Rates (FDRs) for peptide and protein identifications were set to 1% with both platforms. The protein database used was a combination of the reference proteome of *Saccharomyces cerevisiae* (6049 sequences retrieved from UniProtKB release 2020_04, Database_1.fasta) and the sequences of the 48 human proteins included in the UPS1 (https://www.sigmaaldrich.com/content/dam/sigma-aldrich/life-science/proteomics-and-protein/ups1-ups2-sequences.fasta, Database_2.fasta). The files named proteins.txt and proteingroups.txt, generated by PD and MQ, respectively, were used as input for statistical analyses. Proteins not labeled as “IsMasterProtein” were filtered out from the PD file, whereas proteins labeled as “reverse” were filtered out from the MQ file. Protein lists with complete identification and quantification data are provided in Data Set S3.

The protein identification/quantification files generated in this study were deposited to the ProteomeXchange Consortium (http://proteomecentral.proteomexchange.org) via the PRIDE partner repository with data set identifier PXD022169. The original mass spectrometry files were already available in the ProteomeXchange repository with identifiers PXD001819 (main data set) and PXD003472 (second data set).

### Statistical Analyses

Correlation analyses were carried out comparing measured vs expected protein abundance values for each quantitative approach. Proteins quantified in less than half of the points analyzed were filtered out from the analysis inputs. Proteins were grouped into seven categories based on their Spearman’s rank correlation coefficients (ρ), namely: “ρ ≥ 0.95”, “0.90 ≥ ρ > 0.95”, “0.75 ≥ ρ > 0.90”, “0.50 ≥ ρ > 0.75”, “0 ≥ ρ > 0.50”, “ρ < 0” and “too many MVs” (i.e., proteins filtered out due to the high number of MVs), and the distribution of proteins in each category was compared across the six quantification methods considered.

Differential analyses of protein abundance were carried out between sample groups at different protein amounts (three or four replicates per sample). Concerning the main data set, the following comparisons, corresponding to different values of protein abundance log ratio (LR), were evaluated: P1 vs P2 (LR = 1), P1 vs P3 (LR = 2), P1 vs P4 (LR = 3.3, high abundance range), P4 vs P6 (LR = 3.3, intermediate abundance range), P6 vs P9 (LR = 3.3, low-abundance range), P1 vs P9 (LR = 10). The comparison evaluated for the second data set was YH vs Y.

Differential analyses were performed using the Perseus computational platform (version 1.6.7.0),^11^ according to the following steps: (i) data log-transformation: abundance data were subjected to binary logarithmic transformation to approximate a normal distribution, subsequently verified using the Shapiro–Wilk test; (ii) protein filtering: features not reaching 100% valid values in at least one group (for each comparison) were filtered out; (iii) MV replacement: MVs were replaced with a constant value, calculated for each comparison as the binary logarithm of the minimum of the distribution (approximated to the nearest integer) minus 1; (iv) differential analysis: differential protein abundances between groups were tested with a two tails Welch’s *t* test; (v) correction for multiple testing: FDR was calculated based on the *p*-value distribution, according to Benjamini and Hochberg,^12^ considering *q* = 0.05 as the threshold of significance.

A protein abundance LR was also computed as a quantitative measure of the change in abundance of a protein between two sample groups. It was calculated starting from the original abundance data (neither log-transformed nor subjected to MV replacement) as the binary logarithm of the ratio between the mean abundances measured in two sample groups, according to the following formula:
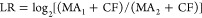
where MA_1_ and MA_2_ are the mean protein abundances measured in group 1 and 2, respectively, while CF indicates the background correction factor. Specifically, a correction factor equal to 1000 (close to the minimum quantitative value measured) was added to eliminate discontinuity due to zero values. The LR was calculated for those proteins which were identified in all replicates of at least one sample group.

On the basis of differential analysis results, we computed the number of true positives (TPs), false positives (FPs), true negatives (TNs), and false negatives (FNs) for each combination of quantification approach and comparison. The criteria used to define TPs, FPs, TNs, and FNs are described in Table S1 (before the application of the LR threshold described below) and Table S2 (after the application of the LR threshold described below). On the basis of these values, we calculated four statistical metrics, namely sensitivity, specificity, accuracy, and precision (the related formulas are provided in Table S3). Balanced accuracy was also computed as the mean between sensitivity and specificity.

Finally, we evaluated the effect of an additional filter based on an LR threshold on the statistical metrics defined above. Specifically, we evaluated the changes in sensitivity, specificity, accuracy, and precision as a function of the LR, ranging between 0 and 25 (in absolute value).

## Results and Discussion

### Experimental Design

**Figure 1:**
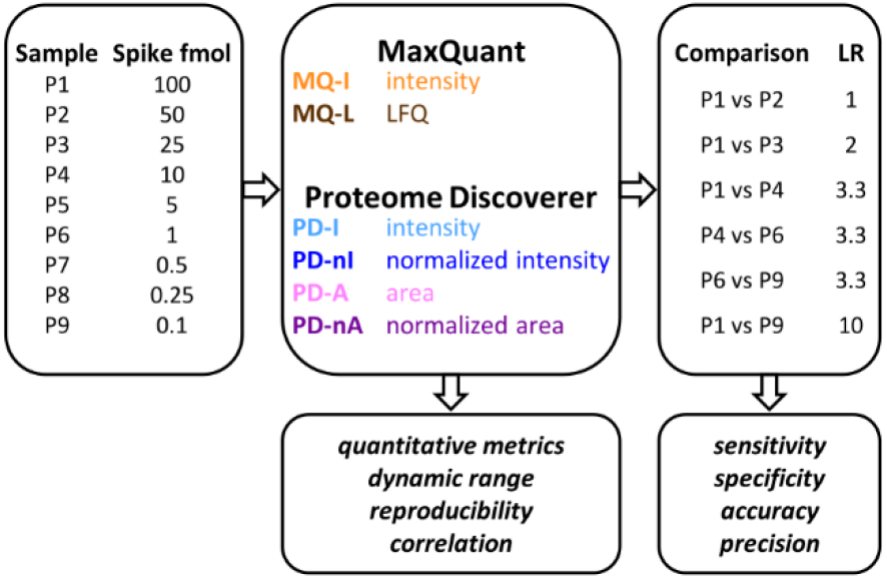
Schematic illustration of the experimental design of the study. The “Spike fmol” column reports the amount of spiked-in proteins contained in the different samples of the main data set, expressed in fmol. The “LR” column lists the protein abundance log ratio between groups expected for the spiked-in proteins in the six sample comparisons evaluated for the main data set.

The experimental design of this study is summarized in Figure 1. The study aims to compare the performances of the label-free protein quantification methods available in the MQ and PD bioinformatic platforms. Six quantitative methods were evaluated, two for MQ and four for PD. MQ analysis returned a (raw) intensity value (MQ-I), corresponding to the peak maximum over the chromatographic profile, for all quantified proteins, as well as a normalized LFQ intensity value (MQ-L) for most of the quantified proteins. PD analysis provided four different quantitative values for each quantified protein: intensity (PD-I), normalized intensity (PD-nI), area (PD-A), and normalized area (PD-nA).

In the main part of the study, the six quantification methods were applied to a publicly available data set,^8^ consisting in nine samples (named P1–P9) run in triplicate. All samples contained a constant background (yeast lysate proteome); a mixture of 48 human proteins was spiked at different amounts in each of the nine samples (with the protein amount analyzed by MS ranging from 100 to 0.1 fmol). The results obtained with the six quantification methods were compared in terms of general quantitative metrics, dynamic range, reproducibility among replicates, as well as correlation between expected and measured values.

Then, the main data set was exploited to simulate a typical differential proteomics scenario, where a small portion of the proteome varies in abundance while most proteins remain constant. Accordingly, six different comparisons were designed to investigate the performance of the quantitative methods with different protein abundance LRs (ranging from 1 to 10) and amounts. The results of the differential proteomic analyses achieved with the six quantification methods were comparatively evaluated according to four different statistical metrics, namely sensitivity, specificity, accuracy, and precision.

Finally, we chose a second data set^9^ containing a “blank” sample (background only, no spiked-in proteins) to further investigate the performance of the six quantification methods. Specifically, two samples were selected from the data set: the first one (YH) containing a human protein standard mixture (25 fmol) spiked in a yeast lysate background, and the second one (Y) containing no spiked-in standards (“blank”). This allowed us to comparatively evaluate the six quantification methods testing the above-mentioned statistical metrics in an extreme scenario.

### Identification and Quantification Metrics, Dynamic Range, and Reproducibility

We initially compared the general performance of MQ and PD in terms of identified and quantified proteins using the main data set. Considering the whole data set, all 48 spiked-in proteins were successfully identified and quantified by MQ and PD, while the overall number of background proteins identified and quantified varied based on the bioinformatic platform used. Specifically, the number of background proteins identified/quantified were 1015/1007 for MQ and 1223/1209 for PD. Identification/quantification metrics in all samples and replicates are provided in Data Set S4.

**Figure 2:**
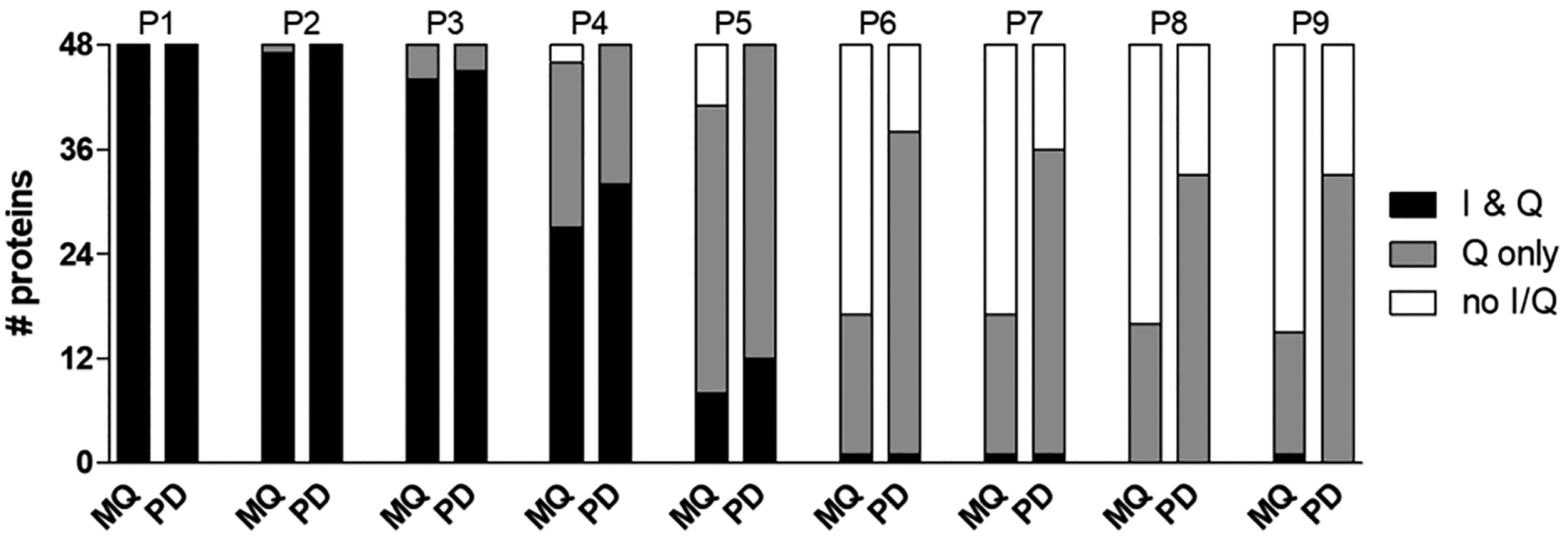
Distribution of the number of spiked-in proteins identified (I) and quantified (Q) by MQ and PD in the nine samples of the main data set. The average number of identified/quantified proteins (*N* = 3 replicate runs) is reported for each sample.

Figure 2 shows how the number of protein identifications and quantifications achieved by the two platforms varies as a function of the spiked-in protein amount. Detailed data at the replicate level are provided in Data Set S5. Although the number of identified proteins was globally comparable between the two platforms, the quantification rate reached by PD from P6 (1 fmol) downward was almost 2-fold compared to that reached by MQ. PD showed a wider dynamic range, as it achieved a quantitative value for over 68% of spiked-in proteins, even at the lowest abundance point (compared to 35% for MQ). The ratio between identified and quantified proteins observed for MQ was comparable to the results of previous studies.^13^

**Figure 3:**
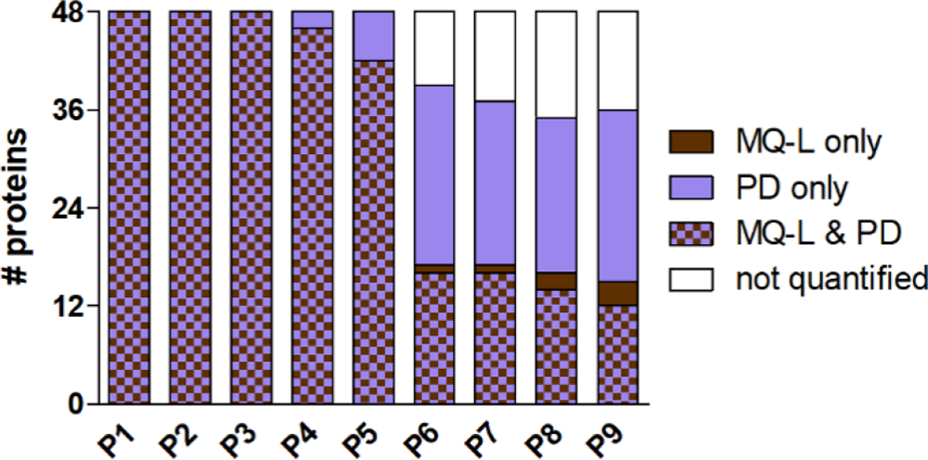
Distribution of the number of spiked-in proteins quantified by MQ-L and/or PD in the nine samples of the main data set. The average number of quantified proteins (*N* = 3 replicate runs) is reported for each sample.

Figure 3 shows the number of spiked-in proteins quantified by both bioinformatic platforms or a single platform (or not quantified at all) in the nine samples. Detailed data at the replicate level (including both MQ-L and MQ-I results, showing few slight differences) are provided in Data Set S6. From P6 downward, most of the quantified spiked-in proteins were either quantified by both platforms or by PD only, while no more than 3 proteins on average were quantified exclusively by MQ.

Also, we comparatively investigated the reproducibility of quantification among sample replicates both in qualitative and in quantitative terms. Under a qualitative perspective, we calculated for each spiked-in protein the number of replicate runs (out of three) per sample where it was consistently quantified. As illustrated in Figure S1, MQ presented a considerably higher number of MVs from P4 (10 fmol) downward, compared to PD. In other words, the probability of quantifying a low-abundance protein in a random run (with that protein being identified in another sample included in the data set) was higher for PD than for MQ. Of note, earlier investigations described a quite high number of MVs reached upon MQ-L analysis, when compared with other quantitative tools.^14,15^ Under a quantitative perspective, we considered the distribution of the abundance values measured by the six methods and calculated their coefficients of variation (CV) among replicates as a measure of variability (and, inversely, of reproducibility). Boxplots in Figure S2, as well as detailed data provided in Data Set S7, show that quantitative values obtained using PD methods (in particular those employing normalization) have a lower CV compared to MQ methods, at least from P1 to P6. A lower CV could be observed in normalization-based methods also when considering background proteins. The CV values measured here for MQ-L quantification were consistent to those reported by other groups in standard protein mixture^16^ and cell line^13^ experiments.

### Correlation between Measured and Expected Quantitative Values

**Figure 4:**
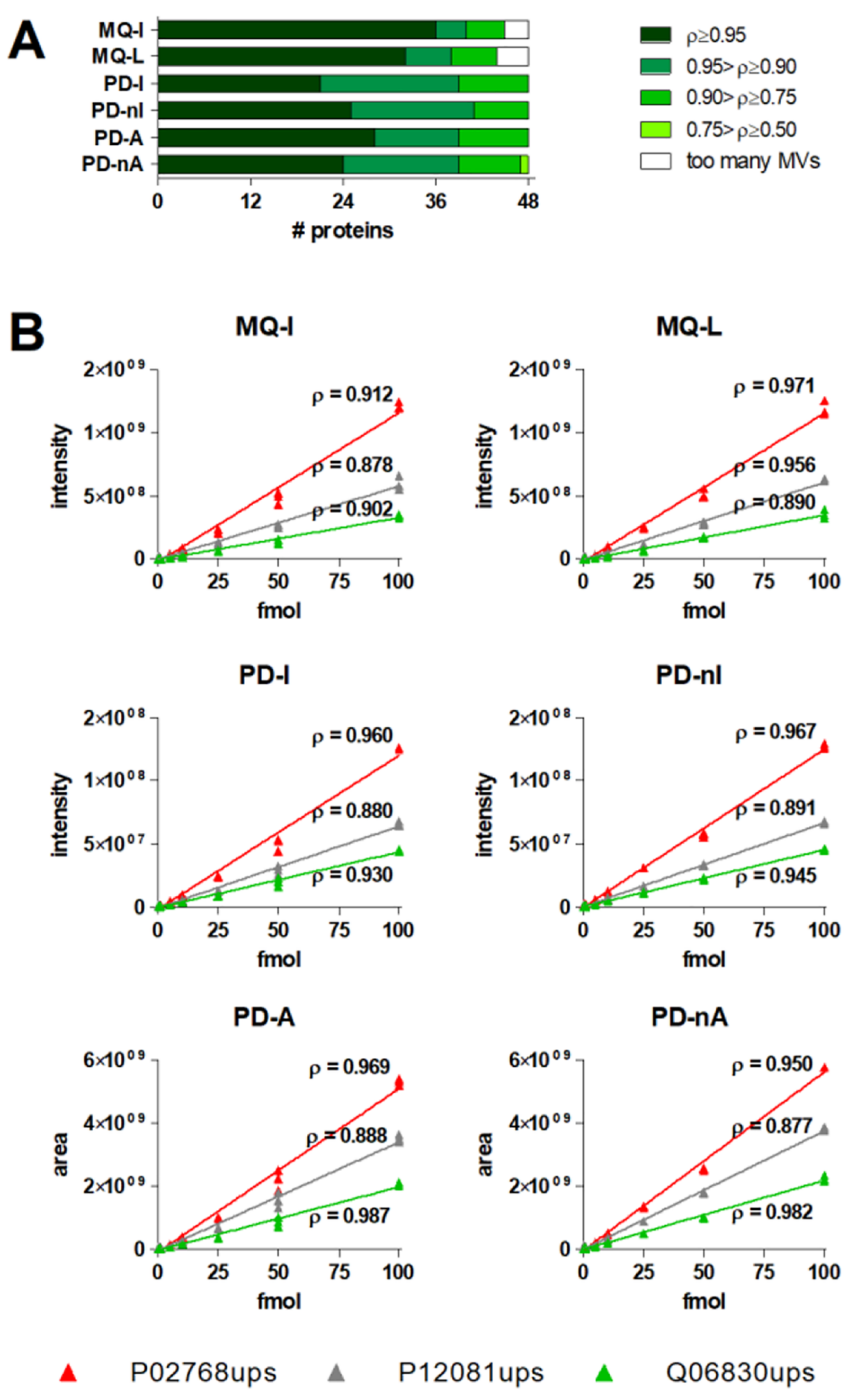
(A) Distribution of spiked-in proteins in classes based on their ρ values, calculated according to Spearman’s correlation between expected and measured quantitative values. Proteins with 14 or more MVs were classified in the “too many MVs” class. (B) Scatterplots of expected (*x*-axis) vs observed (*y*-axis) abundances and regression lines for the three proteins quantified in all sample replicates of the main data set with all quantification methods. Each plot displays the quantification values obtained according to a specific quantification method and reports the ρ value calculated for each protein.

The correlation between measured and expected quantitative values was also calculated (according to Spearman) for the six quantification approaches. The list of the ρ values obtained for each protein of the main data set is reported in Data Set S8. The bar graph in Figure 4A shows the distribution of spiked-in proteins in classes based on their ρ values, revealing that correlation coefficients were globally close to one for all methods. More specifically, MQ-I presented the highest number of proteins with a very strong correlation (ρ ≥ 0.95), followed by MQ-L and PD-A. Instead, PD-nI slightly outperformed the other methods if considering proteins with ρ ≥ 0.90. Figure 4B reports scatterplots (observed vs measured abundances) and regression lines of the three spike-in proteins quantified in all sample replicates with all quantification methods. In all cases, ρ was higher than 0.85. For two proteins out of three, the top ρ was achieved by MQ-L.

We then focused on the low-abundance half of the data set and calculated the correlation between measured and expected quantitative values considering the samples from P5 (5 fmol) downward. As shown in Figure S3A, the global performances of the six methods were clearly poorer compared to those observed for the whole abundance range. No correlation could be calculated for more than 50% of the spiked-in proteins when using MQ methods, as expected, due to a high number of MVs; on the other hand, more than a quarter of the proteins quantified with PD methods exhibited ρ < 0.5. Comparatively speaking, PD-nI and PD-A resulted as the best performing methods in a low-abundance scenario, with PD and MQ methods quantifying around 11 and 24 proteins with ρ > 0.5, respectively. Figure S3B provides a zoom of the scatterplots and regression lines presented in Figure 4B, focusing on the lowest half of the data set. No consistent trends could be found among the three selected proteins. For instance, the measured ρ values were (approximately) 0.7 with MQ-L and 0.3 with PD-A for one protein, while being 0.3 with MQ-L and 0.9 with PD-A for another of the selected proteins.

To summarize, the correlation between measured and expected protein amounts was globally rather good with all quantification methods, although a clear drop in performance could be observed for low-abundance points. In some cases, both quantification tools were not able to detect differences between low signals in a proper way, reaching a low-value plateau; this might be due to background noise or contaminant interference, as seen in other types of protein/peptide measurement by mass spectrometry.^17^

### Statistical Evaluation of the Quantification Methods in a Differential Proteomics Experiment Simulation

In a further investigation, six comparisons between groups were carried out within the main data set to simulate a differential proteomics experimental setting. Each group included three technical replicates of the same sample (i.e., having the same spiked-in protein amount). As illustrated in Figure 1, the expected spiked-in protein abundance LR between groups in the six comparisons ranged from 1 to 10, with three comparisons presenting the same expected LR, but at different abundance ranges.

**Figure 5:**
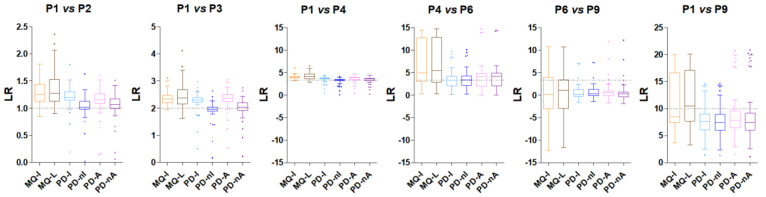
Tukey’s boxplots showing the distribution of spiked-in protein abundance LRs obtained for the six comparisons performed within the main data set using the six quantification methods. The expected LR for each comparison is indicated by the dotted gray line. The LR value was calculated for proteins identified in all replicates of at least one sample group.

Initially, we evaluated the distribution of the observed LR values (in relation to the expected LR values) obtained with the six quantitative methods for the six comparisons (Figure 5). In the P1 vs P2 comparison, we observed an overestimation of LRs for MQ and non-normalized PD methods, while PD-nI provided the best performance. We observed a similar pattern of results both in the P1 vs P3 and P1 vs P4 comparisons, where PD-nI and PD-nA exhibited the lowest deviation between observed and expected LRs, while MQ-L provided the widest variability. In the P4 vs P6 comparison the best LR estimation was reached by PD-I and PD-nI, although the lower abundance range led to an increased variability for all methods (especially for MQ). A significant LR underestimation was observed in the P6 vs P9 comparison, with all values being close to 0, indicating that all quantification methods seem not to work appropriately for low-abundance proteins. Finally, in the P1 vs P9 comparison we saw a substantially increased variability for all methods (probably due to the high amount of MVs in P9); the median LR value measured by MQ-L was the closest to the expected LR value. Considering background proteins (Figure S4), all methods consistently displayed LR values quite close to 0, with non-normalized methods exhibiting a slight overestimation versus a slight underestimation of the normalized ones. In previous comparative studies (not including PD’s Minora among the tools evaluated), MQ-L was found to estimate the expected LR values of spiked-in proteins better than other quantitative methods.^14,15^ This further underlines the accuracy of the estimation reached by Minora in this work. Complete FDR and LR values calculated for each protein, in each comparison and with each method are provided in Data Set S9.

**Figure 6:**
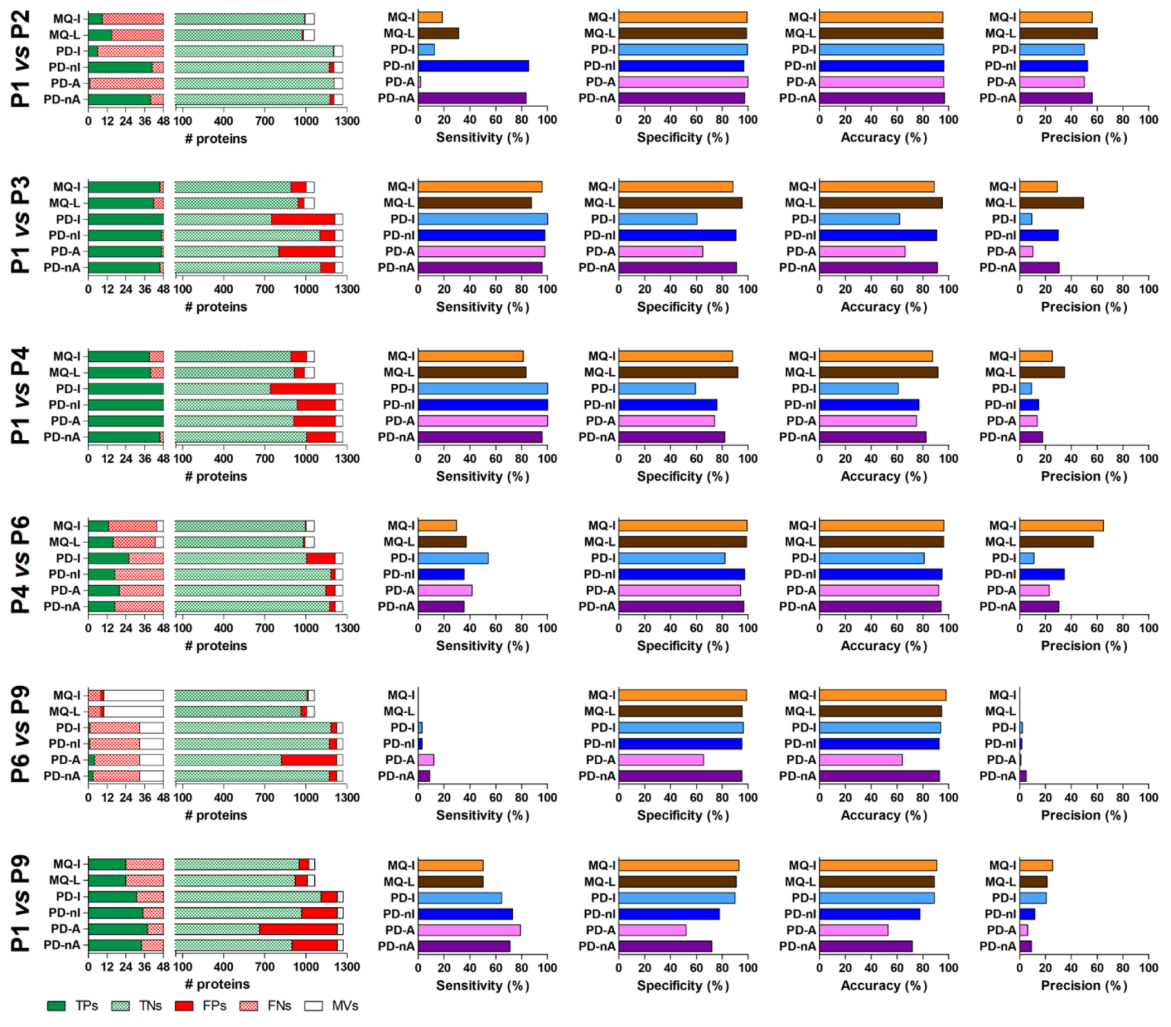
Statistical metrics of differential analysis results obtained from the six comparisons performed with the main data set. Five bar graphs are reported for each comparison. The first bar graph on the left illustrates the distribution of true positives (TPs), true negatives (TNs), false positives (FPs), false negatives (FNs), and proteins filtered out due to the high number of missing values (MVs) for the six quantification methods; spiked-in (left) and background (right) protein data are reported. The remaining four graphs, from left to right, show values of sensitivity, specificity, accuracy, and precision, respectively, reached by the six quantification methods.

Then, we parsed the differential analysis results to calculate the number of TPs, TNs, FPs, and FNs (see Table S1 for details) for each comparison. On the basis of these data, we computed four statistical metrics (namely, sensitivity, specificity, accuracy, and precision; see Table S3 for details) to evaluate the performance of the quantitative methods in a differential proteomics setting. Results are shown in Figure 6.

Considering the P1 vs P2 comparison (narrow LR and high protein abundance), PD-nI and PD-nA clearly outperformed the other methods in terms of sensitivity. Specificity and accuracy were instead comparable among methods, whereas MQ-L showed the higher precision value. With such a narrow LR, the impact of data normalization, at least for PD, appeared to be particularly relevant.

Passing to a LR value of 2 (P1 vs P3 comparison), the results changed considerably. Sensitivity values were much higher than in the previous comparison (almost all spiked-in proteins were correctly identified as differentially abundant), as well as comparable among methods, with a slight preference for PD-I (reaching 100%). On the other hand, a general decrease in specificity and accuracy, and even more in precision, was observed compared to the LR = 1 condition; specifically, MQ-L stood out for specificity, accuracy, and precision. Of note, the dramatic reduction in the precision index was mainly driven by the increased rate of FPs (especially for non-normalized PD methods). These results clearly support the need of an additional filter to reduce FP rate (discussed in detail in the next paragraph).

A similar trend could be measured for the third comparison (P1 vs P4, LR = 3.3), with MQ methods slightly decreasing in sensitivity (mainly due to a higher number of FNs) and normalized PD methods slightly decreasing in the other three metrics (mainly due to a higher number of FPs). Three methods (PD-I, PD-nI, and PD-A) reached 100% sensitivity, while MQ-L outperformed the other methods in terms of specificity (92%), accuracy (92%), and precision (35%).

The next comparison (P4 vs P6) maintained the same LR, but at a lower abundance range. Globally, the number of FNs raised considerably, resulting in a clear decrease in sensitivity. A slight reduction of FPs could also be observed. PD-I was the most sensitive method, while MQ-I reached the maximum for specificity (99%), accuracy (96%), and precision (65%).

A low-abundance range was reached with the fifth comparison (P6 vs P9), keeping LR constant (3.3). The number of TPs and sensitivity dropped to zero (MQ), very low (intensity-based PD methods), and low (area-based PD methods) values. Precision tended to zero as well, whereas the low number of FPs ensured almost unvaried values for specificity and accuracy (except for PD-A) compared to the previous comparison.

The last comparison reached a wider LR (10), corresponding to a remarkable 1000-fold difference in spiked-in protein abundance between the two groups compared. Sensitivity was globally acceptable, with PD-A reaching 79% (against 50% of MQ methods); on the contrary, the lower number of FPs led MQ methods (as well as PD-I) to good levels of specificity and accuracy. The relatively low sensitivity (at least when compared to other comparisons with narrower LRs and involving P1) might be explained by a considerable quantitative variability in the P9 group, related to the presence of MVs (and thus to their imputation), concomitant with the application of a parametric statistical test.

Specificity, sensitivity, and precision values measured in this study after MQ-L analysis were globally in line with previously published results, presented in comparison with those obtained using labeling methods.^16^ Furthermore, a recent study showed that MQ-L outperformed other quantitative tools (not including PD) according to several statistical metrics,^14^ highlighting the value of the results achieved by the approaches compared in this study.

### Increasing Specificity, Accuracy, and Precision Using an Optimized Log Ratio-Based Threshold

Since a relatively high number of FPs were observed in several comparisons (likely due to background noise), we wondered whether the application of an additional LR-based threshold could help reduce FPs and therefore increase specificity, accuracy, and precision of differential analyses. Accordingly, we investigated how the four statistical metrics varied as a function of LR threshold (ranging from 0 to 25). Results are shown in Figure S5 (for detailed data see Data Set S10 and S11; an updated definition of TPs, TNs, FPs, and FNs, as resulting upon application of the LR threshold, is provided in Table S2). As expected, a strong reduction in sensitivity was observed around the expected LR value for each comparison. Instead, a clear increase in specificity was seen as the LR threshold increased, with a higher slope between 0 and 1. Accuracy trend was generally characterized by a steep increase (usually within LR = 1), followed by a plateau and a slight decrease. A Gaussian-like trend of precision value as a function of LR threshold was seen for the first three comparisons, with the maximum close to the expected LR value; the comparisons involving low-abundance points presented more complex and irregular trends, further supporting a poorer quality of measures at lower amounts.

On the basis of these results, we identified (for each quantification method) the LR threshold maximizing each of the statistical metrics evaluated, considering the average value among the six comparisons performed within the main data set (Table S4). Since sensitivity and specificity reached their respective maximum at very low and very high LR values, we considered the maximum of balanced accuracy (mean between sensitivity and specificity) to calculate the LR threshold enabling the best balance between sensitivity and specificity. In order to identify a univocal LR threshold for each quantification method, we computed the mean of the three LR thresholds maximizing, respectively, balanced accuracy, accuracy and precision. Accordingly, the following optimized LR thresholds were set: 1.4 for MQ-I, 1.0 for MQ-L, 1.6 for PD-I, 1.3 for PD-nI, 1.6 for PD-A, and 1.1 for PD-nA.

**Figure 7:**
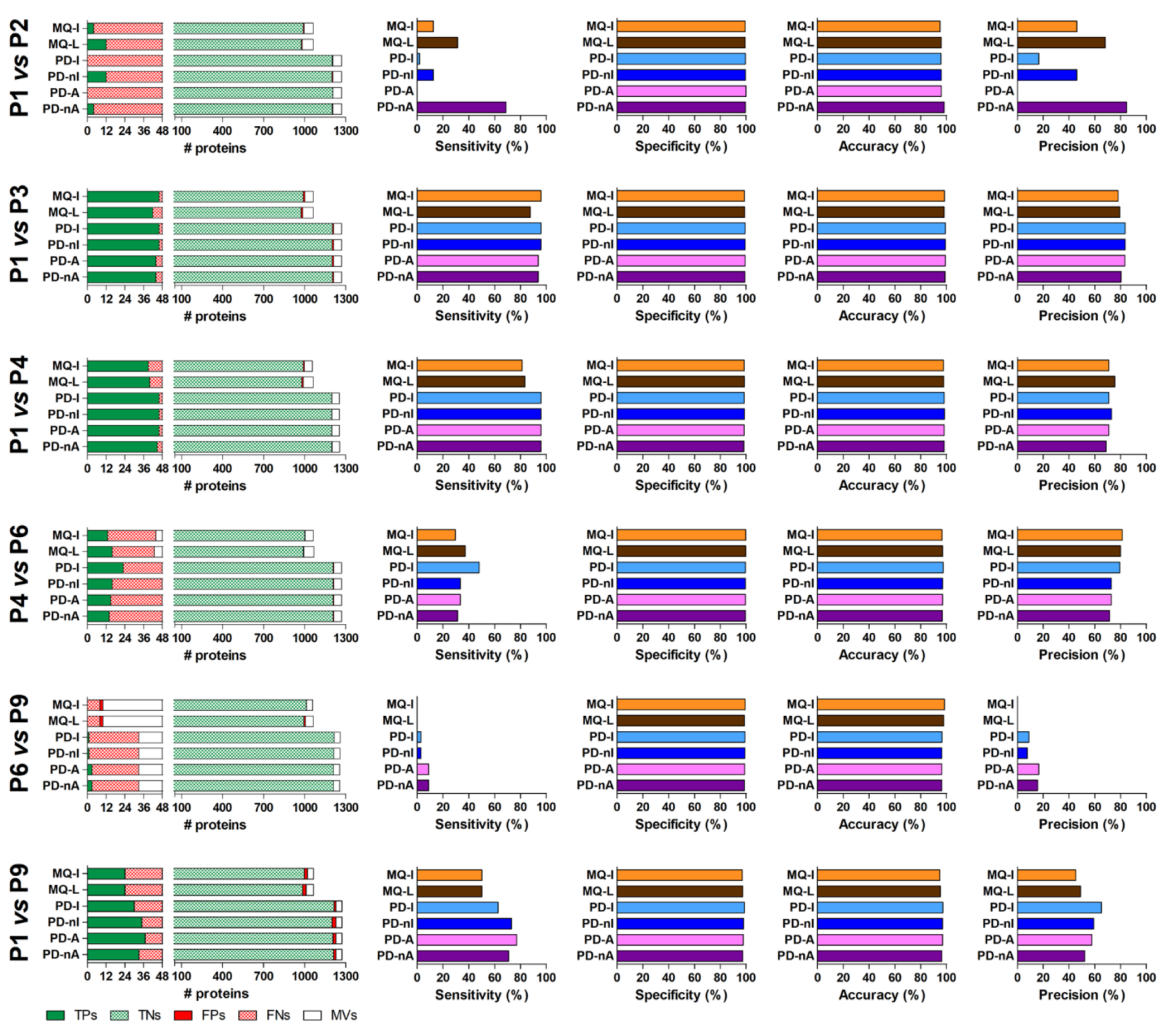
Statistical metrics of differential analysis results obtained from the six comparisons performed with the main data set, upon application of an optimized LR-based threshold. The LR threshold was set to 1.4 for MQ-I, 1.0 for MQ-L, 1.6 for PD-I, 1.3 for PD-nI, 1.6 for PD-A, and 1.1 for PD-nA. The legends for the five bar graphs shown for each comparison are identical with those described for Figure 6.

The values obtained for each statistical measure after the application of the optimized LR-based threshold are shown in Figure 7. The reduction of the number of FPs was clear and generalized, leading to a strong increase in specificity and accuracy (and, to a lesser extent, in precision) at the cost of a very slight loss in sensitivity. Consistently with our strategy, also Ramus and colleagues described an increase of sensitivity and precision upon the application of a z-score-based filter.^8^

### Further Investigation of Quantification Efficiency Using a Data Set with a “Blank” Sample

Finally, we searched for another data set containing a “blank” sample, to further investigate the performance of the six quantification methods in the extreme case in which the abundance of the spiked-in proteins is zero. Within the data set, a sample containing a human protein standard mixture spiked in a yeast lysate background (YH) was reanalyzed along with the “blank” sample (yeast background only, Y).

Initially, we were interested in checking how many human proteins had been quantified in the “blank” sample. Although none of the 48 human standard proteins was expected to be present in sample Y, no less than 21 and 27 of them were quantified on average in the “blank” sample replicates by MQ and PD, respectively. To confirm that these “false” quantifications were related to an incorrect alignment and matching of chromatographic peaks and not to peptide fragmentation and identification, we analyzed the “blank” sample replicates alone (data not shown) and consistently found no human proteins quantified with PD and a single quantification with MQ.

**Figure 8:**
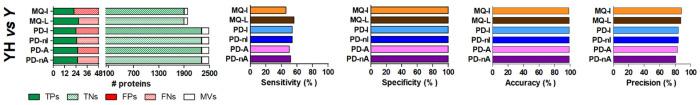
Statistical metrics of differential analysis results obtained from the second data set. The legends for the five bar graphs are identical with those described for Figure 6.

Then, we performed a differential proteomics comparison between YH and Y using the six quantification methods. According to the differential analysis results, very slight differences could be observed among methods (Figure 8). Sensitivity was globally around 50% (the best result was 56% for MQ-L, whereas the worst was 46% for MQ-I), while specificity and accuracy were respectively 100% and 99% for all methods; precision ranged between 81% (PD-nA) and 88% (MQ-I). As noted above for the P1 vs P9 comparison of the main data set, the relatively low sensitivity might be explained by a MV-related variability in the Y group associated with the application of parametric statistics.

Upon the application of the LR thresholds set for the main data set (described in the previous paragraph), a slight improvement in precision was seen (specificity and accuracy were already excellent without applying any LR-based filter), as illustrated in Figure S6. Complete results referring to the second data set are available in Data Set S12.

In summary, the use of a data set containing a “blank” sample revealed that both MQ and PD (with the latter providing worse results) can detect a relatively high number of “false” quantitative values when dealing with low/zero-abundance proteins, suggesting possible biases in chromatographic peak alignment and mapping with peptide sequences. We cannot rule out that this might be partially due to interference of human peptide traces from previous LC-MS runs. Anyhow, this issue did not have a clear impact on differential analysis performance, since specificity, accuracy, and precision values were excellent, while sensitivity was comparable with the results obtained with the main data set when considering similar comparisons.

## Conclusions

This study presents a comparative evaluation of six different label-free approaches for protein quantification, available in the PD and MQ suites. The overall performance of the Minora quantification tool, embedded in the PD bioinformatic platform, was at least comparable to that achievable using the established MQ suite, especially when combined with data normalization. More specifically, the following final considerations can be made on the basis of the comparisons described above:

- PD outperformed MQ in terms of quantification yield, dynamic range, and reproducibility.
- All methods exhibited a good correlation between measured and expected protein amounts, at least considering medium/high-abundance points.
- PD methods including normalization were the most accurate in estimating the protein abundance LR between groups after differential analysis and displayed a higher sensitivity when comparing groups with a narrow LR.
- MQ methods generally reached slightly higher specificity, accuracy, and precision values.
- Normalized approaches were globally more specific, accurate, and precise compared to the corresponding non-normalized ones.
- Applying an optimized LR-based threshold led to a considerable increase in specificity, accuracy, and precision, with a very slight loss in sensitivity.
- When dealing with low-abundance proteins, the quality of measurements reached by both platforms was in some cases quite poor, suggesting that features with a high number of MVs or an average low abundance might be discarded during data preprocessing, in order to reduce the number of statistical tests and relax FDR correction for multiple testing.

Taken together, our data provide useful indications for scientists interested in applying an MS1-based label-free protein quantification method to their studies.

## Supporting Information Available

Supplementary Figures and Tables: Distribution of the number of spiked-in proteins quantified by MQ and PD in the nine samples of the main data set, based on the number of replicate runs in which proteins were quantified (Figure S1); Tukey’s boxplots showing the distribution of the CV of the abundance values obtained for each sample of the main data set with the six quantification methods (Figure S2); Distribution of spiked-in proteins in classes based on their ρ values, calculated according to Spearman’s correlation between expected and measured quantitative values (samples from P5 downward of the main data set); Scatterplots of expected (*x*-axis) vs observed (*y*-axis) abundances and regression lines for the three proteins quantified in all sample replicates with all quantification methods (samples from P5 downward of the main data set) (Figure S3); Tukey’s boxplots showing the distribution of background protein abundance LRs obtained with the six quantification methods in the six comparisons performed with the main data set (Figure S4); Variation of statistical metrics (sensitivity, specificity, accuracy, and precision) as a function of the LR threshold, based on the differential analysis results obtained with the six quantification methods in the six comparisons performed with the main data set (Figure S5); Statistical metrics of differential analysis results obtained from the second data set, upon application of an optimized LR-based threshold (Figure S6); Definition of TPs, FPs, TNs, and FNs (Table S1); Definition of TPs, FPs, TNs, and FNs upon application of an optimized LR-based threshold (Table S2); Formulas of the statistical metrics calculated in this study (Table S3); LR values corresponding to the maximum of the statistical metrics calculated based on the differential results obtained applying the six quantification methods to the main data set (mean of the six comparisons) (Table S4) (PDF)
Data Set S1: Mass spectrometry analysis parameters (XLSX)
Data Set S2: Protein identification and quantification parameters used in the MQ and PD workflows (XLSX)
Data Set S3: Overall protein identification and quantification data obtained with the main data set (XLSX)
Data Set S4: Number and CV of quantitative values measured for each protein of the main data set using the six quantification approaches (XLSX)
Data Set S5: Number of proteins identified and/or quantified in each replicate and in each sample (average and cumulative values) of the main data set using the six quantification approaches (XLSX)
Data Set S6: Number of proteins identified and/or quantified by MQ-I, MQ-L, and PD in each sample replicate of the main data set (XLSX)
Data Set S7: Number of proteins quantified by MQ-I, MQ-L, and/or PD in each sample replicate of the main data set (XLSX)
Data Set S8: Spearman’s rank correlation coefficients obtained for each protein of the main data set with the six quantification approaches (XLSX)
Data Set S9: FDR and LR values calculated for each protein in the six comparisons performed with the main data set, based on the differential analysis results obtained with the six quantification approaches (XLSX)
Data Set S10: Variation of TPs, TNs, FPs, and FNs as a function of the LR threshold for the six comparisons performed with the main data set, based on the differential analysis results obtained with the six quantification approaches (XLSX)
Data Set S11: Variation of sensitivity, specificity, accuracy, balanced accuracy, and precision as a function of the LR threshold for the six comparisons performed with the main data set, based on the differential analysis results obtained with the six quantification approaches (XLSX)
Data Set S12: Identification, quantification, differential analysis, and statistical metrics data obtained with the second data set (XLSX)
