# Supplementary material for: Comparative Evaluation of MaxQuant and Proteome Discoverer MS1-Based Protein Quantification Tools
Source: J Proteome Res. 2021 May 26;20(7):3497–507. doi: 10.1021/acs.jproteome.1c00143 (PMC8280745; doi:10.1021/acs.jproteome.1c00143)
Supplement: Supplementary file 1 — pr1c00143_si_001.pdf [file pr1c00143_si_001.pdf]

Comparative Evaluation of MaxQuant and Proteome Discoverer MS1-based Protein Quantification Tools

Antonio Palomba[1], Marcello Abbondio[2], Giovanni Fiorito[2,3], Sergio Uzzau[2], Daniela Pagnozzi[1], and Alessandro Tanca[2]*

[1]*Porto Conte Ricerche, Loc. Tramariglio, 07041 Alghero, Italy*
[2]*Department of Biomedical Sciences, University of Sassari, Viale San Pietro 43/B, 07100 Sassari, Italy*
[3]*MRC Centre for Environment and Health, Imperial College London, Norfolk Place, W2 1PG London, UK*

Supplementary Figures and Tables

Figure S1: Distribution of the number of spiked-in proteins quantified by MaxQuant (MQ) and Proteome Discoverer (PD) in the nine samples of the main dataset, based on the number of replicate runs (N from 0 to 3) in which proteins were quantified.
Figure S2: Tukey's boxplots showing the distribution of the coefficients of variation (CV) of the abundance values obtained for each sample of the main dataset with the six quantification methods.
Figure S3: **A**. Distribution of spiked-in proteins in classes based on their ρ values, calculated according to Spearman's correlation between expected and measured quantitative values. Samples from P5 downward of the main dataset are included in the graph. Proteins with 8 or more missing values (MVs) were classified in the "too many MVs" class. **B**. Scatterplots of expected (x-axis) *vs* observed (y-axis) abundances and regression lines for the three proteins quantified in all sample replicates with all quantification methods. Samples from P5 downward of the main dataset are included in the graph. Each plot displays the quantification values obtained according to a specific quantification method and reports the ρ value calculated for each protein.
Figure S4: Tukey's boxplots showing the distribution of background protein abundance log ratios (LRs) obtained with the six quantification methods in the six comparisons performed with the main dataset.
Figure S5: Variation of statistical metrics (sensitivity, specificity, accuracy, and precision) as a function of the protein abundance log ratio (LR) threshold, based on the differential analysis results obtained with the six quantification methods in the six comparisons performed with the main dataset.
Figure S6. Statistical metrics of differential analysis results obtained from the second dataset, upon application of an optimized protein abundance log ratio (LR)-based threshold. The LR threshold was set for each method according to the results obtained with the main dataset (see Figure 7 in the manuscript for details). The first bar graph on the left illustrates the distribution of true positives (TPs), true negatives (TNs) false positives (FPs), false negatives (FNs) and proteins filtered out due to the high number of missing values (MVs) for the six quantification methods; spiked-in (left) and background (right) protein data are reported. The remaining four graphs, from left to right, show values of sensitivity, specificity, accuracy, and precision, respectively, reached by the six quantification methods.
Table S1: Definition of true positives (TPs), false positives (FPs), true negatives (TNs), and false negatives (FNs).
Table S2: Definition of true positives (TPs), false positives (FPs), true negatives (TNs), and false negatives (FNs), upon application of an optimized log ratio (LR)-based threshold.
Table S3: Formulas of the statistical metrics calculated in this study.
Table S4: Log ratio (LR) values corresponding to the maximum of the statistical metrics calculated based on the differential results obtained applying the six quantification methods to the main dataset (mean of the six comparisons).

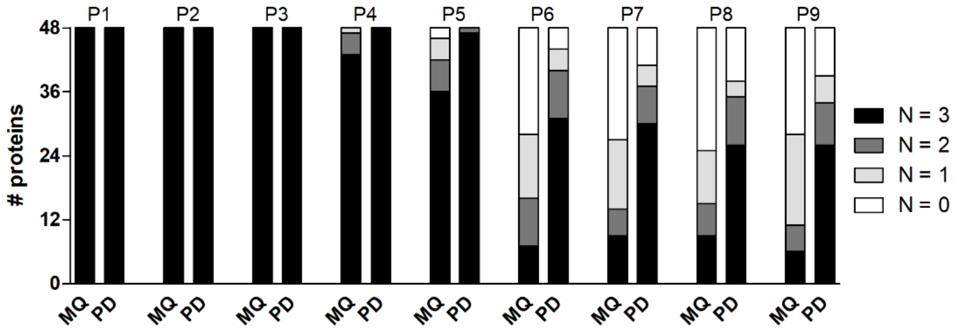

Figure S1. Distribution of the number of spiked-in proteins quantified by MaxQuant (MQ) and Proteome Discoverer (PD) in the nine samples of the main dataset, based on the number of replicate runs (N from 0 to 3) in which proteins were quantified.

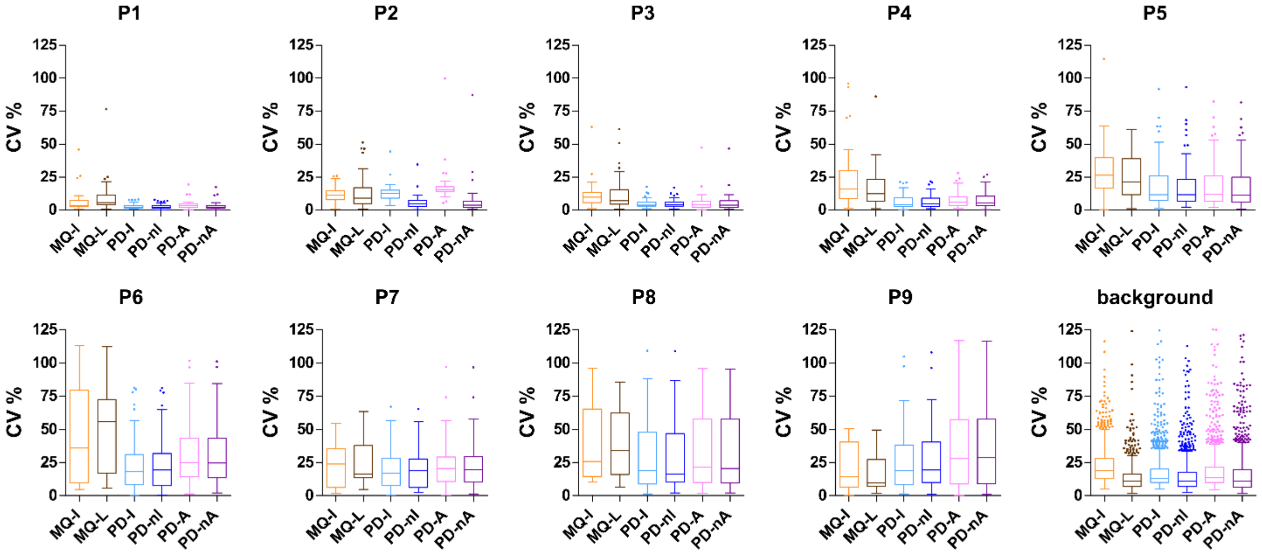

Figure S2. Tukey's boxplots showing the distribution of the coefficients of variation (CV) of the abundance values obtained for each sample of the main dataset with the six quantification methods.

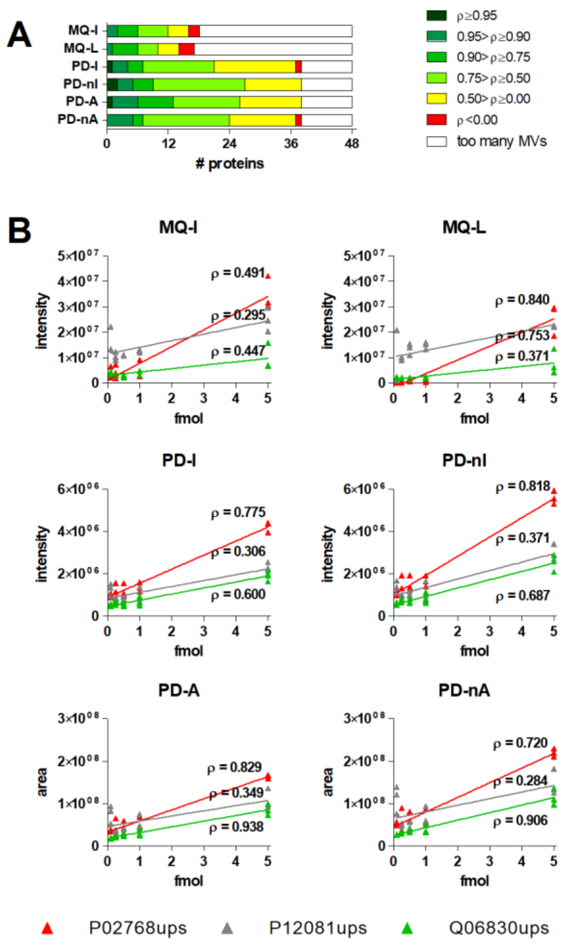

Figure S3. **A**. Distribution of spiked-in proteins in classes based on their ρ values, calculated according to Spearman's correlation between expected and measured quantitative values. Samples from P5 downward of the main dataset are included in the graph. Proteins with 8 or more missing values (MVs) were classified in the "too many MVs" class. **B**. Scatterplots of expected (x-axis) *vs* observed (y-axis) abundances and regression lines for the three proteins quantified in all sample replicates with all quantification methods. Samples from P5 downward of the main dataset are included in the graph. Each plot displays the quantification values obtained according to a specific quantification method and reports the ρ value calculated for each protein.

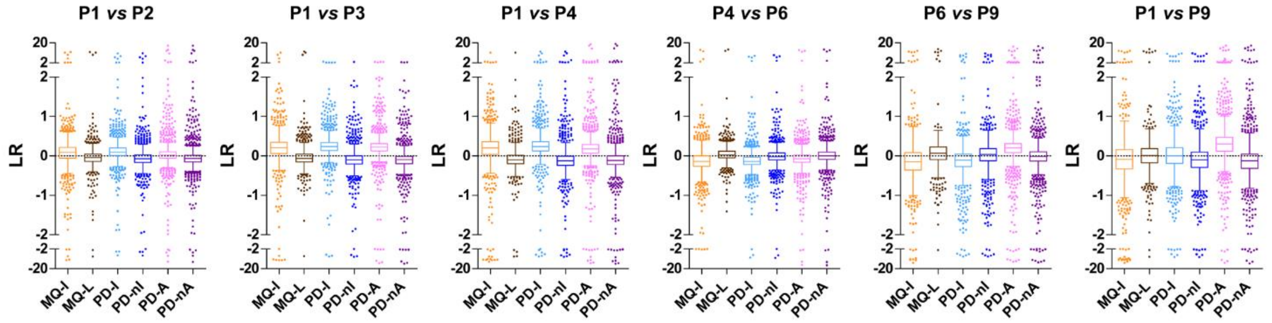

Figure S4. Tukey's boxplots showing the distribution of background protein abundance log ratios (LRs) obtained with the six quantification methods in the six comparisons performed with the main dataset.

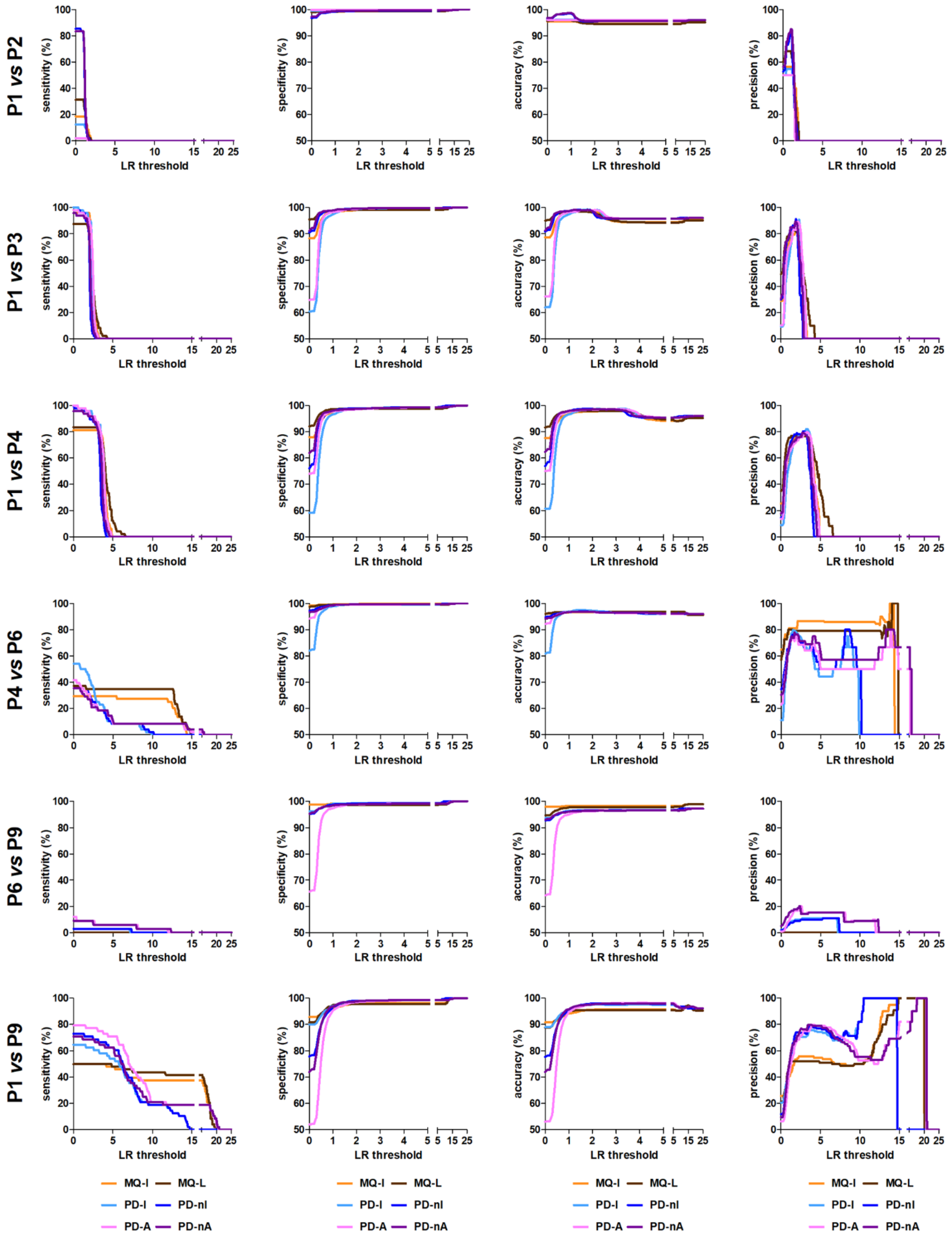

Figure S5. Variation of statistical metrics (sensitivity, specificity, accuracy, and precision) as a function of the protein abundance log ratio (LR) threshold, based on the differential analysis results obtained with the six quantification methods in the six comparisons performed with the main dataset.

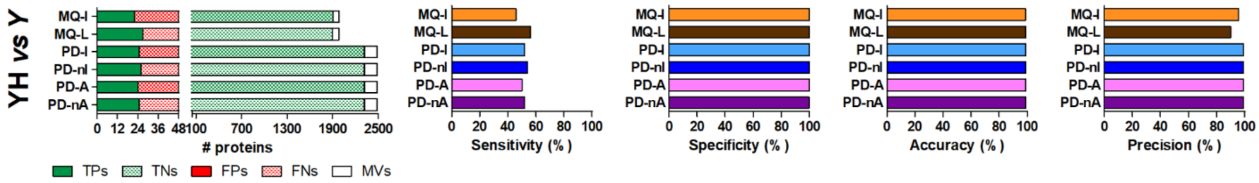

Figure S6. Statistical metrics of differential analysis results obtained from the second dataset, upon application of an optimized protein abundance log ratio (LR)-based threshold. The LR threshold was set for each method according to the results obtained with the main dataset (see Figure 7 in the manuscript for details). The first bar graph on the left illustrates the distribution of true positives (TPs), true negatives (TNs) false positives (FPs), false negatives (FNs) and proteins filtered out due to the high number of missing values (MVs) for the six quantification methods; spiked-in (left) and background (right) protein data are reported. The remaining four graphs, from left to right, show values of sensitivity, specificity, accuracy, and precision, respectively, reached by the six quantification methods.

Table S1. Definition of true positives (TPs), false positives (FPs), true negatives (TNs), and false negatives (FNs).

**Outcome**	**Condition to be respected**		
	**Spike/Background**	**FDR <0.05**	**LR**
TPs	Spike	Yes	Positive
TNs	Background	No	Positive
			Negative
FPs	Spike	Yes	Negative
	Background		Positive
			Negative
FNs	Spike	No	Positive
			Negative

Table S2. Definition of true positives (TPs), false positives (FPs), true negatives (TNs), and false negatives (FNs), upon application of an optimized LR-based threshold.

**Outcome**	**Condition to be respected**			
	**Spike/Background**	**FDR <0.05**	**LR**	**LR threshold**
TPs	Spike	Yes	Positive	Passed
TNs	Background	No	Positive	Passed
			Negative	
		Yes	Positive	Not passed
			Negative	
		No	Positive	
			Negative	
FPs	Spike	Yes	Negative	Passed
	Background		Positive	
			Negative	
FNs	Spike	No	Positive	Passed
			Negative	
		Yes	Positive	Not passed
			Negative	
		No	Positive	
			Negative	

Table S3. Formulas of the statistical metrics calculated in this study.

Statistical metrics	Formulas
Sensitivity (True Positive Rate)	TPs/(TPs + FNs)
Specificity (True Negative Rate)	TNs/(TNs + FPs)
Accuracy	(TPs + TNs)/(TPs + TNs+ FPs + FNs)
Balanced accuracy	(Sensitivity + Specificity)/2
Precision (Positive Predictive Value)	TPs/(TPs+FPs)

Table S4. Log ratio (LR) values corresponding to the maximum of the statistical metrics calculated based on the differential results obtained applying the six quantification methods to the main dataset (mean of the six comparisons).

**Statistical metrics**	**MQ-I**		**MQ-L**		**PD-I**		**PD-nI**		**PD-A**		**PD-nA**	
	**Max**	**LR**	**Max**	**LR**	**Max**	**LR**	**Max**	**LR**	**Max**	**LR**	**Max**	**LR**
Sensitivity	45.9%	0.0	48.2%	0.0	55.7%	0.0	65.8%	0.0	55.5%	0.0	65.1%	0.0
Specificity	100.0%	14.9	100.0%	14.9	100.0%	12.1	100.0%	12.2	100.0%	18.9	100.0%	19.0
Balanced accuracy	72.2%	1.2	73.5%	1.0	76.3%	1.1	81.5%	0.8	75.8%	1.2	81.4%	1.0
Accuracy	97.0%	1.8	97.0%	1.0	97.5%	2.2	97.4%	1.9	97.4%	2.1	97.4%	1.1
Precision	54.0%	1.2	58.6%	1.0	56.0%	1.4	59.1%	1.1	55.7%	1.4	62.2%	1.1
